# Electromagnetic Energy Absorption in a Head Approaching a Radiofrequency Identification (RFID) Reader Operating at 13.56 MHz in Users of Hearing Implants Versus Non-Users

**DOI:** 10.3390/s19173724

**Published:** 2019-08-28

**Authors:** Patryk Zradziński, Jolanta Karpowicz, Krzysztof Gryz

**Affiliations:** Laboratory of Electromagnetic Hazards, Central Institute for Labour Protection—National Research Institute (CIOP-PIB), Czerniakowska 16, 00-701 Warszawa, Poland

**Keywords:** acoustic sensor, numerical modeling, electromagnetic field, specific energy absorption rate (SAR), bone conduction, environmental engineering, public health

## Abstract

The aim of this study was to model the absorption in the head of an electromagnetic field (EMF) emitted by a radiofrequency identification reader operating at a frequency of 13.56 MHz (recognized as an RFID HF reader), with respect to the direct biophysical effects evaluated by the specific absorption rate (SAR), averaged over the entire head or locally, over any 10 g of tissues. The exposure effects were compared between the head of a user of a hearing implant with an acoustic sensor and a person without such an implant, used as a referenced case. The RFID HF reader, such as is used in shops or libraries, was modeled as a loop antenna (35 × 35 cm). SAR was calculated in a multi-layer ellipsoidal model of the head—with or without models of hearing implants of two types: Bonebridge (MED-EL, Austria) or bone anchored hearing aid attract (BAHA) (Cochlear, Sweden). Relative SAR values were calculated as the ratio between the SAR in the head of the implant user and the non-user. It was found that the use of BAHA hearing implants increased the effects of 13.56 MHz EMF exposure in the head in comparison to non-user—up to 2.1 times higher localized SAR in the worst case exposure scenario, and it is statistically significant higher than when Bonebridge implants are used (Kruscal–Wallis test with Bonferroni correction, p < 0.017). The evaluation of EMF exposure from an RFID reader with respect to limits established for the implant non-user population may be insufficient to protect an implant user when exposure approaches these limits, but the significant difference between exposure effects in users of various types of implants need to be considered.

## 1. Introduction

Conductive hearing loss can, in various cases, be compensated for by bone conduction hearing implants of the Bonebridge or bone anchored hearing aid attract (BAHA) types (implanted behind the earlobe), which use sound waves conducted through the skull bones directly to the inner ear, where they are perceived in the same way as natural sound. They are electronic devices consisting, among other things, of an acoustic sensor, an audio processor, other electronics, a system of wireless trans-skin signal transmission, magnets, a vibrating system, and titanium elements screwed to the skull [1,2,3,4,5]. The mentioned implants work in different ways. In the Bonebridge type implant, the sounds recorded by the acoustic sensors (outer part of the implant) are converted into an electrical signal. This is transmitted to the demodulator (inner part of implant) via transmission coils by electromagnetic induction. The electromagnetic induction is also used to transfer the energy necessary to operate the implanted part of the device. The demodulator then converts the signals and transmits them to the BC–FMT (bone conduction–floating mass transducer), which uses vibrations to stimulate the internal ear’s auditory system, where they are perceived as natural sound [5,6]. On the other hand, in the BAHA type implants, the sounds recorded by the acoustic sensor are processed into vibrations of the sound processor’s magnet and are transmitted to the implanted part. It causes the skull bones to vibrate, etc., as described for the Bonebridge implant [4,6].

### 1.1. RFID Readers and EMF Exposure Nearby

During their work, but also in their daily life, users of hearing implants may experience exposure to electromagnetic fields (EMF) from various sources in various locations against their body (examples of electrical devices being quite common sources of EMF in the everyday environment include: induction hobs, microwave ovens, wireless communication or identification devices, welders, high voltage transmission lines, and so on). We studied in detail the influence on the users of hearing implants caused by EMF emitted by radiofrequency identification (RFID) devices using EMF from the frequency band 1–30 MHz, recognized as high frequency RFID (RFID HF) and known to be sources of significant EMF exposure near their antennas, the use of which is still very popular [7,8]. Other RFID devices use EMF from the frequency bands: 30–300 kHz, recognized as low frequency (RFID LF); 860–960 MHz, recognized as ultra-high frequency (RFID UHF) and 2.400–2.4835 GHz, recognized as super high frequency (RFID SHF).

RFID devices are used for various purposes, for example aimed at the identification, monitoring, controlling or managing of objects in libraries, shops, enterprises, medical centers (medical devices, pharmaceuticals, biological samples, even patients), and so on; the marking of animals, prisoners, or dangerous substances to control them in the public environment; contactless payment (cards, public transport cards, toll collection on highways and bridges); in near-field communication devices, and in access control to various buildings or rooms. From the RFID technologies available on the market, various RFID HF systems are used in principle in contactless payment cards, ticketing, and in control systems in shops, libraries, offices, and medical centers.

RFID devices are composed of the reader and TAGs (electronic elements that are exchanging data with RFID readers through radio waves) attached to the objects under control or monitoring [7]. Readers of RFID HF devices are frame (loop) transmitting/receiving antennas of various shape and dimensions. TAGs are small devices that emit a signal using electricity from their battery (active TAGs), or using energy absorbed from the EMF reader emission (passive TAGs). To detect the presence of an RFID passive labeled object, i.e., the object to which passive TAG is attached, the EMF signal from TAG needs to be strong enough to induce a usable electric signal in the reader’s antenna. Because the signal induced in the RFID reader is stronger when its antenna is larger, larger antennas are more sensitive to signals from TAGs located in larger spaces around the reader, which can make identification faster and easier. The “reading range” of a particular device is the maximum distance between the reader and a TAG, where a TAG can still be “found”. This maximum “reading range” depends on the TAG design and reader sensitivity, though the current settings of a device may shorten it according to the needs of the user (lowering the level of emitted EMF).

International standards provide characteristics of the reading ranges and emitted EMF of various RFID HF technologies using passive TAGs (i.e., TAGs that are useful even over many years, much longer than in the case of active TAGs, whose operation depends on the life of batteries). The mentioned reading range is linked to the strength of the magnetic field emitted from the reader, which has to be able to successfully charge (energize) a TAG and to allow wireless contact between the reader and the TAG. The small antenna, short-range (SR–SA) readers used with proximity cards require a magnetic field exceeding 1.5 A/m affecting a TAG located at a distance from the reader equal to the reading range—up to several centimeters (ISO/IEC 14443-2:2016 standard) [8]. The medium-range readers, usually equipped with a medium or large antenna (MR–MA or MR–LA, respectively) require a field of 0.15 A/m at a distance equal to the reading range—up to 1.5 m in that case (ISO/IEC 15693-1:2010 standard) [9].

Currently, various designs of RFID HF readers are available on the market (with different antenna dimensions and shapes, and different maximum emitted power), for various purposes and conditions of use. For example, in the offer of leading manufactures (FEIG Electronic, 3M, Andea Electronics, metraTec), the following offers can be found [10,11,12,13]:

(a) Short-range readers with small antennas (SR–SA) (dimensions in millimeters: 40 × 30, 78 × 78, 84 × 84, 100 × 100, 120 × 75, etc.) used in contactless payment cards, ticketing, document tracking in offices, access control, time attendance, controlling and management of samples with biological material in medical centers, and typically characterized by an emitted power up to 0.2 W,
(b) Medium-range readers with medium size antennas (MR–MA) (dimensions in millimeters: 220 × 180, 310 × 310, 320 × 200, 340 × 250, 350 × 350, 380 × 290, 390 × 360, 400 × 400, etc.) used in gates, as a desktop, or fixed to walls or furniture in shops, libraries, industry, offices (document tracking), and medical centers (controlling and management of samples with biological material, even patients), and typically characterized by an emitted power up to 8 W,
(c) Medium-range readers with large antennas (MR–LA) (dimensions in millimeters: 520 × 420, 830 × 610, etc.), used in gates or fixed to walls in restaurants (controlling food delivery), logistics, and industry, and typically characterized by an emitted power up to 30 W.

**Figure 1:**
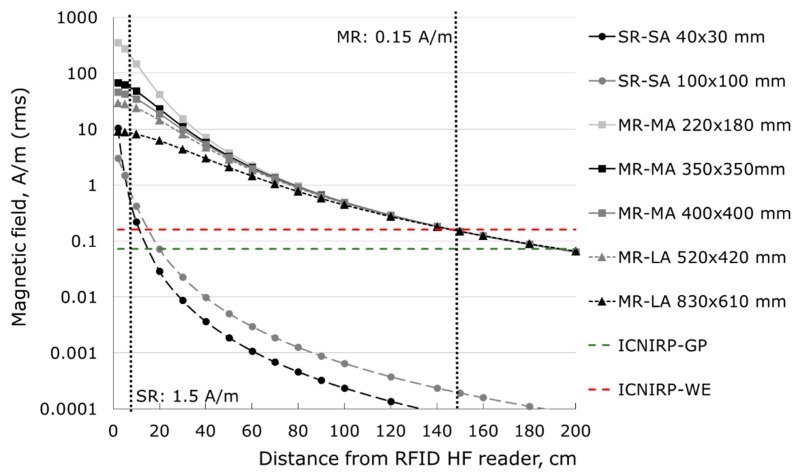
Electromagnetic field (EMF) distribution (magnetic component) in the function of distance from high frequency radiofrequency identification (RFID HF) readers with antennas of various dimensions set at the maximum reading range, along the axis of the reader (SR—short-range reader with a maximum reading range of 5 cm, MR—medium-range reader with a maximum reading range of 1.5 m, SA—small size antenna, MA—medium size antenna, LA—large size antenna, International Commission on Non-Ionizing Radiation Protection (ICNIRP)–GP—magnetic field limit of general public exposure, ICNIRP–WE—magnetic field limit of workers exposure).

The EMF continuously emitted by the reader searching passive TAGs increases rapidly at shorter distances to the antenna—at least several times higher exposure can be expected in their vicinity than at a distance equal to the reading range. Figure 1 shows the spatial distributions of the magnetic field in the vicinity of RFID HF readers of various dimensions, when the operation mode (i.e., EMF emission) was set at the maximum reading range (as defined by ISO/IEC 14443-2:2016 and ISO/IEC 15693-1:2010 standards), which was compared with human exposure limits provided by the International Commission on Non-Ionizing Radiation Protection (ICNIRP). In the vicinity of RFID HF readers set at the maximum reading range, EMF exposure exceeds ICNIRP limits regarding exposure to a magnetic field set at flat level in the frequency range 10–400 MHz: 0.16 A/m of workers exposure (ICNIRP-WE) and 0.073 A/m of general public exposure (ICNIRP-GP) [14]—up to approximately 20 cm from the SR–SA only, but up to approximately 2 m from the MR–MA readers. ICNIRP guidelines include the note that provided exposure limits may not necessarily preclude effects on implant users.

Today, the most popular RFID HF system uses an EMF at a frequency of 13.56 MHz and operates with a medium reading range set within the distance of up to 1.5 m from the reader [7,8,9]. Taking into account the characteristics of EMF emission discussed above (Figure 1), medium-sized readers need particular attention in the discussed context of human exposure to EMF, e.g., in shops or libraries, where humans are typically present in their vicinity for extended periods. RFID HF readers may be fixed to furniture, such as tables at various heights (on or below), special gates or walls (on or covered inside)—and in this case the TAG’s labeled objects are moved into the reader. The other popular option is the use of manually movable readers that are moved by the operator to be close to the scanned objects in order to test whether they are labeled by RFID TAG.

Summarizing, the reader may be located in any direction and distance from the human body—intentionally or unintentionally—and in some cases the exposure to EMF emitted by it may be prolonged, especially in the case of workers, and the exposure level compared or exceeding ICNIRP limits.

The effects of exposure to EMF emitted by such readers should be evaluated with respect to workers and the public (e.g., customers) and are dependent on the reader size, set reading range (i.e., operation mode) and location against the body. In any of these cases, the exposed person may use hearing implants.

### 1.2. Principles of Protection Against the Effects of EMF Exposure

In brief, the general principle of evaluating safety during exposure to EMF established that, in the case of exposure exceeding the limits provided for the electric and magnetic field strength, e.g., the above mentioned ICNIRP limits, the metrics of direct biophysical effects in the body need to be evaluated—the electric field induced in the body by low frequency EMF, or heat induced by high frequency EMF. The direct biophysical effect of exposure to EMF of frequencies exceeding 100 kHz (including the 13.56 MHz frequency of EMF emitted by the considered RFID HF reader) is tissue heating caused by the absorption of EMF energy. The metric widely used to evaluate this exposure effect is the specific energy absorption rate (SAR), expressed in watts per kilogram (W/kg)—usually evaluated using numerical simulations and averaged over the entire body, or locally over any 10 g of tissues, and averaged over the six-minute exposure duration.

Due to the possible location of an RFID HF reader close to the human body and EMF exposure exceeding exposure limits up to even approximately 2 m, it is necessary to evaluate SAR values from the EMF influence. Legislation and guidelines on the evaluation of the EMF exposure of medical implant users claim to evaluate to what extent the effects of exposure in their body may be stronger because implanted objects may significantly change the spatial distribution of the electric field and currents induced in the body [15]. To compare the effects from EMF exposure between the body of a hearing implant user and a person without an implant (non-user), the relative SAR values are usually used—e.g., calculated as the ratio between the SAR in the head of an hearing implant user and the SAR in the head of a non-user [14,16]. There are many papers discussing the relative effects of EMF exposure in various cases of humans who are different from the regular persons considered in the regular compliance tests performed because of legal requirements [17,18,19]. This approach does not involve any consideration of the EMF exposure changes in time—because both people are counted to be exposed for the same duration. As mentioned above, especially in the case of workers, prolonged exposure near an RFID reader may occur. For the case of other persons, e.g., customers, a typical exposure scenario involves a short stay near a reader, e.g., at a shop cash desk or library reception desk, though even customers may need several minutes to complete their business and will stay within the continuously emitted EMF near the reader searching for passive TAGs.

In the case of evaluating the effects of exposure to EMF in implant users, the possible interference with electronic components of implants also needs to be taken into consideration. It is usually evaluated following the principles of electromagnetic compatibility tests and is not considered in this work. In the considered high frequency range, a second kind of possible effect in the structures of implants that may raise their temperature through induced currents are negligible (though, in case EMF exposure at low frequency, such heating may need to be taken into account) [17,19].

### 1.3. The Aim

The aim of this study was to model the absorption in the head of an EMF at a frequency of 13.56 MHz, emitted by an RFID HF reader used with passive TAGs, with respect to the rules established for the evaluation of the SAR values from EMF exposure, and to compare the effects between the user of Bonebridge (MED-EL, Austria) or BAHA (Cochlear, Sweden) type implants and a person without implants (non-user).

The effects of exposure to EMF in tissues adjacent to hearing implants have been already tested using numerical models of exposure to low frequency EMF from medical devices. In that case it was found that the strength of the electric field induced in tissues adjacent to the implant was up to 4.5-times higher in the case of a BAHA implant user (and up to 3.4-times higher in the case of a Bonebridge implant user), in comparison to the values obtained in these tissues in a person without an implant [1,2]. Additionally, the results of this studies showed that the relative effects of exposure to low frequency EMF in implant users depends on the spatial distribution of the EMF influencing the tissues adjacent to the implant, which is the function of the EMF source location against the implant. This experience justified the studies focused on exposures of users of such implants to EMF at higher frequencies, where other metrics of exposure effects (SAR) are applied. An example of such an exposure scenario discussed in this paper is the EMF exposure near an RFID HF reader continuously emitting an EMF of 13.56 MHz frequency to energize and search passive TAGs.

There are no reports in the literature about the level of relative effects of EMF exposure from RFID HF readers concerning users of bone conduction hearing implants. The results of our study support the evaluation of the medical implant users’ exposure to EMF from the low megahertz range, covered by European mandatory requirements regarding worker protection, as well as non-binding requirements from the European recommendation regarding general public protection, as also advised by international guidelines from ICNIRP [14,16,20].

## 2. Materials and Methods

### 2.1. Numerical Simulations

The simulations of SAR in the numerical model of a head of an implant user exposed to EMF emitted by the RFID HF reader were carried out by CST Studio Suite (CST, Germany) software, with a microwave package based on the finite integration technique (FIT), and time domain solver with simulation accuracy of −60 dB. Open (add space) boundary conditions were used on all walls of the calculation domain located 60 cm from the nearest surface of model of a head of an implant user or model of RFID HF reader. Numerical models were composed of 12–14 million voxels and the resolution of the numerical model of an implant user of 1.8–2.0 mm had the finest resolution of 0.3 mm, set in the vicinity of the implants.

The estimated uncertainty of numerical simulations ±25% (K = 1) was within the range accepted by international standards and is comparable to the uncertainty of the published results of numerical simulations on RFID readers [21,22,23,24].

The SAR values were calculated as averaged over the whole head, or averaged over any 10 g of continuous tissue (localized) [14,16,21]. Localized SAR values were obtained using the algorithm according to IEC/IEEE 62704-1:2017 [21]. The absorption in the head of an EMF emitted by an RFID HF reader was modeled with respect to the rules established for the evaluation of SAR values—the resolution of the human body model better than 1/15 of the wavelength in tissues as required by the relevant standard IEC 62232-2011 [25].

### 2.2. EMF Source

A pilot test carried out for RFID HF readers with a small antenna (with dimensions 12 cm × 7.5 cm) and a medium-size antenna (with dimensions 35 cm × 35 cm) set at their maximum reading ranges showed up to 70-times lower localized SAR values in cases of readers with a small antenna (the typical maximum reading range up to several cm) in comparison to SAR values evaluated from EMF emitted by a medium-size antenna (with a maximum reading range up to 1.5 m). A second pilot test compared the relative SAR near the medium-size antenna in direct proximity (2 cm away) and at distances of 10, 50, and 100 cm away from the head, and provided confirmation that in more heterogeneous EMF closer to the reader antenna, the relative localized SAR is higher (2.07, 1.88, 1.48, and 1.30 respectively).

Based on the results of tests, the most common medium-size RFID readers were considered for this study—RFID HF (high frequency) operating at 13.56 MHz with a reading range of up to 1.5 m. The modeled RFID HF reader was a medium-size loop emitting antenna with dimensions of 35 cm × 35 cm (with a typical size and a symmetric shape). Such antennas (MR–MA) are used for example in shops and libraries (for example, in desktop readers, placed on a desk, table, or assembled below a table top, or set in anti-thief gates or as manually movable reader). This model of reader has been chosen because its dimensions are in the middle of MR–MA dimensions and EMF field distribution in its vicinity may also represent exposure near other common MR–MA readers, and even near MR–LA which are used less frequently (Figure 1). In addition, as indicated in the pilot studies the effects of exposure to EMF emitted from MR–MA readers are many times higher in comparison to effects from short-range, small-antenna (SR–SA) readers. Medium-range, large-antenna (MR–LA) readers have not been covered in detail by our investigations due to the fact that they are very rarely used.

The numerical model of the RFID HF reader was validated by comparing the magnetic and electric field distribution near the reader, measured (uncertainty ±20%, K = 1) by an EMF meter (type NBM-550 from Narda STS, Germany, with an electric field probe EF0391, frequency range: 100 kHz −3 GHz; measurement range: 0.2–320 V/m and magnetic field probe HF 3061, frequency range: 300 kHz–30 MHz; measurement range: 0.017–16 A/m) and numerically simulated (uncertainty ±25%, K = 1) by CST Studio Suite 2016 (Computer Simulation Technology, Germany) software.

### 2.3. Exposure Scenarios

**Figure 2:**
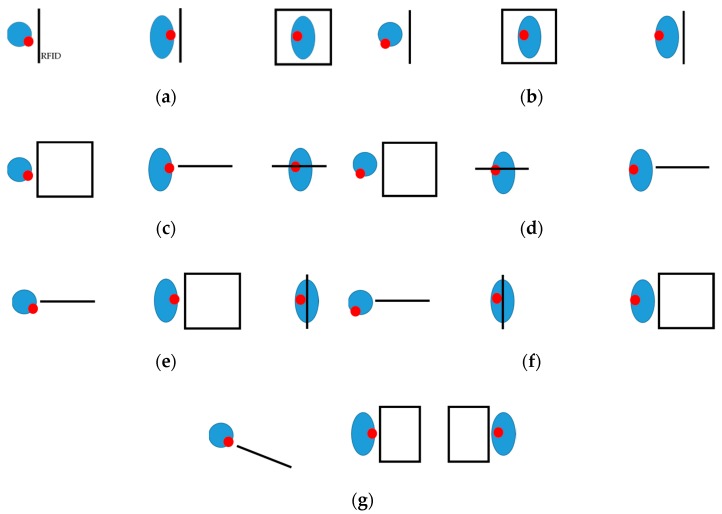
Projections of investigated exposure scenarios with the RFID HF reader: (**a**) on the implanted side, parallel to sagittal cross-sections of the model of the head; (**b**) in front of the hearing implant user’s face, parallel to frontal cross-sections of the model of the head; (**c**) on the implanted side, parallel to transversal cross-sections of model of the head; (**d**) in front of the hearing implant user’s face, parallel to transversal cross-sections of model of the head; (**e**) on the implanted side, perpendicular to sagittal cross-sections of the model of the head; (**f**) in front of the hearing implant user’s face, perpendicular to frontal cross-sections of the model of the head and (**g**) on the implanted side, at an angle of 25 degrees to frontal cross-sections of the model of the head (reader plane perpendicular to the plane of the internal part of the hearing implant fixed to the skull bone), (red dot—implant location, shown on the transversal, frontal, and sagittal cross-sections of the head, in each exposure scenario respectively).

In general, the exposure scenarios used in the study considered that the RFID HF reader is located on the implanted side of the head (scenarios: a, c, and e) or in front of the hearing implant user’s face (scenarios b, d, and f) at a distance of 2 cm to the closest surface of the model of the head. At a distance of 2 cm, the exposure to EMF is more heterogeneous, and so a higher relative SAR (SAR increase in the implant user in comparison to a person without an implant) than at longer distances is expected (Figure 1 and Figure 2), as shown also in pilot tests. Over longer distances, the EMF distribution is more and more homogeneous and it has a smaller relative effect on SAR in implant users. The position of the plane of the investigated RFID HF reader was: parallel to sagittal (vertical, front to back) cross-sections of the model of the head (scenario a), parallel to frontal (vertical, side to side) cross-sections of the model of the head (scenario b), parallel to transversal (horizontal, side to side) cross-sections of model of the head (scenarios c and d), perpendicular to sagittal cross-sections of the model of the head (scenario e), perpendicular to frontal cross-sections of the model of the head (scenario f) and at an angle of 25 degrees to frontal cross-sections of the model of the head (reader plane perpendicular to the plane of the internal part of the hearing implant fixed to the skull bone), (scenario: g) (Figure 2). The variety of exposure scenarios were tested as required by European directive 2014/53/EU (RED directive), to cover “all intended operating conditions and the reasonably foreseeable conditions” [26]. The considered exposure scenarios represent various polarizations and the spatial distribution of EMF affecting the hearing implant user, providing broad coverage of realistic exposure scenarios near RFID HF readers or other sources of EMF of a similar frequency and spatial distribution, for example physiotherapeutic applicators.

### 2.4. Implant Models

Investigations were carried out using numerical models of structures of two types of bone conduction hearing implants: Bonebridge (MED-EL, Austria) and bone anchored hearing aid (BAHA) (Cochlear, Sweden) [1,2,3,4,5]. In the numerical models of both implants, the dimensions, shape, and material parameters (conductivity and relative permittivity) were taken into account for elements of both the external and the internal parts. The conductivity (relative permittivity) of the particular elements of implants are:

- copper—6 × 10^7^ S/m (1.0)—sound processor (external), transmission coils (eternal/internal—Bonebridge only)
- steel—7 × 10^6^ S/m (1.0)—magnets (external)
- titanium alloy—5.6 × 10^5^ S/m (1.0)—element fixed to bone (internal)
- silicone—2.5 × 10^−4^ S/m (11.9)—cover of Bonebridge implant (internal)
- polyethylene terephthalate (PET)—1 × 10^−15^ S/m (3.5)—housing of sound processor and magnet (external).

### 2.5. Model of the Head of Implant User 

The head of the hearing implant user or non-user was modeled as a multi-layer ellipsoid with dimensions corresponding to the dimensions of the 5th, 50th, and 95th percentile male head of the Polish population [1,2,27]. The thicknesses of particular tissue layers were the median values of people being implanted with hearing implants: 4 mm for skin, 4 mm for fat, and 9 mm for skull bone. The inner part of the model corresponded to brain tissue. The relative permittivity (based on Gabriel’s data) of particular layers was: skin—177.1, fat—11.83, bone—59.3, and brain (average value of white and gray matters)—208.3 while the conductivity was: 0.384 S/m, 0.030 S/m, 0.128 S/m, and 0.252 S/m, respectively [28].

### 2.6. Evaluation of SAR Values from EMF Exposure 

The absorption in the head of an EMF emitted by an RFID HF reader at a frequency of 13.56 MHz was modeled and SAR values calculated. The EMF exposure effects resulting from the presence of a hearing implant were compared to the effects for a person without an implant (non-user) exposed under the same conditions. This comparison was presented as the ratio (KI) between the SAR values calculated in the user’s tissues next to the implant, SAR(impl), and the SAR values in the tissues of a non-user, SAR(nu): KI = SAR(impl)/SAR(nu), analyzed for each type of hearing implant separately.

The results of our analysis are useful in order to make the users of hearing implants (and other active medical implants) aware of the scale of the increase in EMF exposure effects near implants. The KI parameter may also be applied to approximate the direct effects in hearing implant users exposed near other EMF sources of a structure similar to the modeled MR–MA RFID HF reader, even when the dimensions or frequency of the emitted EMF slightly differ. In contrast, the absolute SAR values are highly sensitive to the parameters of the exposure scenario, such as the dimensions of the EMF source and the distance to the EMF source defining the polarization and spatial distribution of the EMF affecting humans, as well as the level of exposure, which depends on the emitted EMF level (i.e., the actual settings of the reading range of the considered RFID HF reader) and the distance to the EMF source. Moreover, in various countries it is required (by legislation or guidelines) from the manufacturers of devices to evaluate SAR caused in a regular person by exposure to EMF sources (e.g., European Union directives—Europe, Federal Communication Commission (FCC)—United States of America, Regulatory Compliance Mark (RCM)—Australia [16,29]) and to provide the results of this evaluation in the manuals for the use of this device, or to label the devices as compliant with EMF exposure guidelines (such as the European “CE” mark). Because of the case sensitivity of the absolute SAR values, and because of the availability of SAR-related information in the devices’ manuals, the absolute SAR values obtained in our models were not presented in this paper, as they are applicable only for the modeled individual case of exposure situation.

Based on the coverage of the broad variability of EMF exposure scenarios representing cases where the highest influence on the SAR values is expected (explained in Section 2.3), we also believe that the maximum value of relative SAR obtained following our work plan may be considered as the worst case of this parameter, representing an increase in EMF hazards for hearing implant users exposed near various RFID HF readers or similar sources of HF EMF.

### 2.7. Statistical Analysis

A parametrical r-Pearson’s test was used for the analysis of relations between the distribution of the magnetic and electric component of EMF emitted by an RFID HF reader achieved by measurements and simulations (boundary condition—significance level: p = 0.05). The power of correlation was assessed by applying the typically used four-step criterion for the r-values: no correlation (|r| < 0.2); weak correlation (0.2 < |r| < 0.4); mean correlation (0.4 < |r| < 0.7) and strong correlation (|r|> 0.7).

The significance of differences between the KI ratio (between the SAR values calculated in the Bonebridge or BAHA implant user’s tissues next to the implant SAR(impl), and the SAR values in the tissues of a non-user SAR(nu)) in the considered numerical model of a head of various dimensions and under various exposure scenarios were tested using the ANOVA Kruskal–Wallis non-parametric test for independent groups (boundary condition—significance level: p = 0.05) with a Bonferroni correction for multiple comparison (boundary condition—significance level: p = 0.05/3 = 0.017, because the number of analyzed parameters is 3: dimensions of the model of the head of an implant user, implant type, and exposure scenario). The statistical analysis was carried out using STATISTICA 9.0 software (StatSoft, Palo Alto, CA, USA).

## 3. Results and Discussion

**Figure 3:**
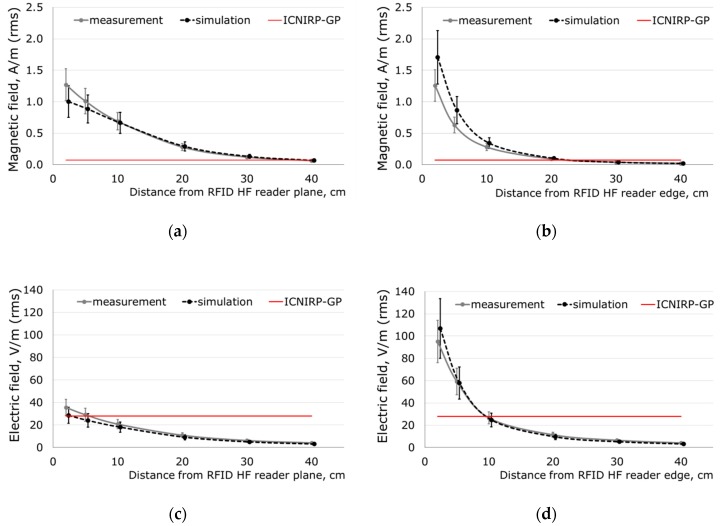
The EMF distribution in the function of distance from an RFID HF reader with a reading range of 30 cm measured by NBM 550 meter and simulated by CST Studio Suite: magnetic field along the axis perpendicular to the reader plane (**a**) and (**b**) along the axis perpendicular to the reader edge, (**c**) electric field along the axis perpendicular to the reader plane, and (**d**) along the axis perpendicular to the reader edge (whiskers—range of values due to uncertainty of measurements or simulations (K = 1); ICNIRP–GP—reference level (limit) for general public exposure; distance from an RFID reader to the center of the measurement probe; results at 2 cm and 5 cm away were extrapolated on the basis of measurements at 10–40 cm away from the reader.

The numerical model of the EMF source was validated by measurements of the electric and magnetic field distribution in the vicinity of the single RFID HF reader with a reading range of 30 cm. At a distance next to an RFID HF reader, where the EMF distribution is highly heterogeneous and the level of exposure may exceed exposure limits (Figure 1) and where the considered head of an implant user was located, EMFs were characterized by the results of measurements. Figure 3a,b shows magnetic field distribution along the axis perpendicular to the reader plane (magnetic field along this axis determines the reading range) and along the axis perpendicular to the reader edge (on the side of the reader), respectively. Similarly Figure 3c,d shows electric field distribution. Measurement results were obtained by the volume measurement probe (spatially averaging measured EMF) and simulations by “virtual volume probe”, the central point of both probes were located at particular distances of 10, 20, 30, and 40 cm. The electric and magnetic field at distances of 2 cm and 5 cm were extrapolated from the results of measurements performed in longer distances (measurements of several cm away from the reader were not possible because of measurement probe’s volume of several centimeters). The whiskers on Figure 3 show the estimated uncertainty of measurements: ±20%, (K = 1) and numerical simulations: ±25% (K = 1). The obtained results were compliant within an uncertainty of measurements and numerical simulations (Figure 3a–d). A correlation analysis showed statistically significant strong correlations between EMF distribution in the vicinity of the RFID HF reader obtained by measurements and simulations:

- For magnetic field—Pearson’s r = 0.9541 (strong correlations for |r| > 0.7), statistically significant p = 0.000001 (boundary condition p = 0.05)
- For electric field—Pearson’s r = 0.9934 (strong correlations for |r| > 0.7), statistically significant p < 0.000001 (boundary condition p = 0.05).

In the investigated exposure scenarios, the model of the implanted user’s head is located at a distance of 2 cm from the reader. The length of the sagittal axis of the numerical models of the head used in the investigations was in the range of 18 up to 20 cm. The head occupies an area from 2 cm up to 20–22 cm from the reader. In this range, magnetic field strength values exceed reference level values provided by the ICNIRP for general public exposure evaluation (ICNIRP RL GP = 0.073 A/m) near the RFID HF reader with a reading range of 30 cm (Figure 3a,b). The electric field strength values also exceed ICNIRP RL GP (28 V/m), but only at distances <10 cm (Figure 3d).

Taking into consideration the above mentioned strong exposure to EMF near the investigated RFID HF reader, the immunity of electronic components of bone conduction hearing implants (acoustic sensors and other electronic parts) needs separate consideration, following the previously mentioned electromagnetic compatibility tests.

**Figure 4:**
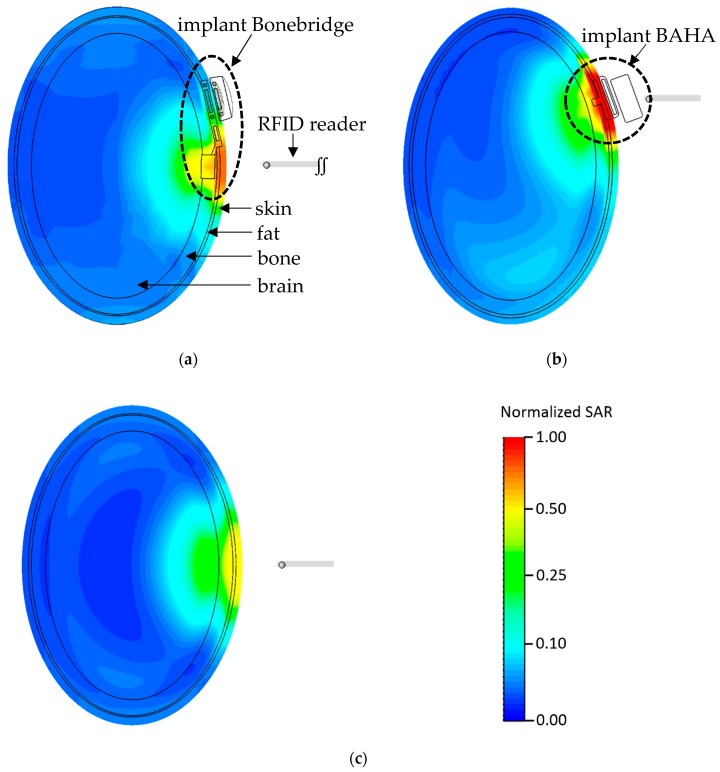
The distribution of localized SAR (at frontal cross-section through the center of the implant part fixed to the bones of the skull) simulated by CST Studio Suite, under exposure to EMF from an RFID HF reader (13.56 MHz) in exposure scenario c, as shown in Figure 2: (**a**) near a Bonebridge implant, (**b**) near a BAHA implant and (**c**) in a non-user (unified normalized logarithmic color scale of absolute SAR values—reference value: maximum localized SAR value calculated for exposure scenario c and BAHA implant user).

The comparison of SAR inside the head of a hearing implant user and a non-user in the case of exposure to high frequency EMF is presented in Figure 4, and evaluated in Table 1 (SAR averaged over the whole head—SARwh) and Table 2 (localized SAR averaged over any 10 g of tissues in head—SARlh). SAR values evaluated in Table 1 and Table 2 are not absolute values. Absolute values depend on emitted field level associated with above mentioned reading range and time of exposure. In a previous study it was found, for example, that SAR values may exceed the general public ICNIRP level when the regular person is present in the vicinity (at a distance of 5 cm) of a medium-size RFID HF reader with a reading range exceeding 100 cm [30].

No changes were found related to the presence of a bone conduction hearing implant on the whole head averaged SAR (SARwh) in comparison to the values in a person without an implant exposed to an EMF at a frequency of 13.56 MHz, emitted by an RFID HF reader (KIwh: 0.99–1.11) independently of the head dimensions and exposure scenarios (Table 1). The changes from the Bonebridge and BAHA type implant in SARwh were found to be within the mentioned uncertainty of numerical simulations ±25% (K = 1). KIwh differences related to the dimensions of the model of the head of an implant user were evaluated as being not statistically significant by the Kruskal–Wallis test with Bonferroni correction, p = 0.027 (boundary condition p = 0.017). Similarly, in relation to implant type or exposure scenario, KIwh differences were also not statistically significant, p = 0.373 and p = 0.056, respectively. This may be explained by the small size of the hearing implant in relation to the volume of the entire head—the presence of the implant changes SAR values only in the tissues adjacent to the implant, which consist only of a small percentage of the volume of the entire head.

The highest differences (KIlh = 2.07) between localized SAR, averaged over any 10 g of tissues in the head (SARlh) of bone conduction hearing implant user’s and head of non-user was found in skull tissue in the model of BAHA implant user head corresponding to the 5th percentile dimensions when the RFID HF reader is located in front of the face of implant user, perpendicular to frontal cross-sections of the model of the head (exposure scenario f, as shown at Figure 2) (Table 2). In the case of a Bonebridge type implant, the corresponding difference was 1.47.

The highest influence on the SARlh value (KIlh = 1.60) of the Bonebridge type hearing implant was found in skull tissue in the model of an implant user’s head corresponding to the 5th percentile dimensions when the RFID HF reader is located at the implanted side, at an angle of 25 degrees to frontal cross-sections of model of the head (exposure scenario g). In the case of the BAHA type implant, the corresponding value was 1.72.

Differences in SARlh (KIlh) obtained for exposure cases when the RFID HF reader was located at the implanted side of the model of the hearing implant user’s head (exposure scenarios a, e, and g) were up to 40% higher and up to 25% lower (exposure scenario c) in comparison to impacts from the reader located in front of the face of hearing implant user’s head (exposure scenarios b, f, and d). KIlh differences related to the exposure scenario were evaluated as being statistically significant by the Kruskal–Wallis test with Bonferroni correction, p = 0.010 (boundary condition p = 0.017).

In the case of exposure scenarios a, b, d, f, and g, differences in SARlh (KIlh) obtained for the hearing implant user’s head of the 5th percentile dimensions were up to 5% and 18% higher in comparison to differences for head of the 50th and 95th percentile dimensions, while in the case of exposure scenarios c and e, values of KIlh were lower by 6% and 12%. The differences were found to be within the mentioned uncertainty of numerical simulations ±25% (K = 1). KIlh differences related to dimensions of the model of the head of an implant user were evaluated as being not statistically significant by the Kruskal–Wallis test with Bonferroni correction, p = 0.430 (boundary condition p = 0.017).

In the case of the BAHA type hearing implant, the differences (KIlh) were up to 50% higher than in the case of the Bonebridge type, independent of exposure case and head dimensions. KIwh differences related to the implant type were evaluated as being statistically significant by the Kruskal–Wallis test with Bonferroni correction, p = 0.0001 (boundary condition p = 0.017).

The obtained results showed that the EMF exposure effects depend (statistically significant) on the location of the RFID HF reader against the implanted side of the head, as well as implant construction and location inside the head model. The SARlh values obtained in the head of bone conduction hearing implant users are higher in comparison to values obtained in a person without an implant, mainly due to the metallic elements of their construction. As a result of technological development, hearing implants are being developed to be more and more resistant to EMF influence on their electronic structure, but they still contain relatively big metal elements which may cause stronger direct effects from EMF exposure in the user’s tissue. For both types of hearing implants, Bonebridge and BAHA, the highest case of exposure effects was found in the exposure scenarios where the RFID HF reader is in front of the face and parallel to transversal cross-sections of the model of the head or is located at an angle of 25 degree to frontal cross-sections of the model of the head, respectively (c and g scenarios). In all these scenarios, the maximum SARlh occurred in the vicinity of the implants and were higher than the maximum SARlh found in the model of a non-user.

SARlh values obtained in the head of BAHA type hearing implant users are higher than in the head of Bonebridge type implant users due to the bigger volume of implanted metallic elements. The obtained results showed that effects of EMF exposure in the head of a bone conduction hearing implant users are comparable to those of non-users during 1.5-times lower EMF exposure (corresponding to the fact that SAR values are proportional to the square of field levels). Taking into account that EMF near an RFID HF reader with a reading range of 30 cm exceed reference level values provided by the ICNIRP for general public exposure evaluation (ICNIRP magnetic field RL GP = 0.073 A/m and electric field RL GP (28 V/m)), as mentioned at Figure 3, the distance where EMF exposure of hearing implants users should be considered to be non-compliant with ICNIRP is also longer. At the EMF exposure equal to the workers limits, SAR in the head of implant user reached 4-times higher values than for exposure equal to limits for general public, which is significantly higher than the expected uncertainty of evaluation of EMF hazards, and because of this attention to EMF exposure effects have to be addressed in the safety programs, especially in case of exposure situations when it may continue over minutes or longer.

It also means that near an RFID HF reader with a reading range of 30 cm or longer, EMF exceeds the levels provided for the evaluation of safety for the use of electronic implants manufactured for use on the EU market (recommended in Appendix A of European standard EN 50527-1:2016), i.e., implants (including bone conduction hearing implants) manufactured to not be disturbed by EMF at a level within the general public exposure limits provided by European Council recommendation 1999/519/EC (based on upper mentioned ICNIRP RL GP) [15,20].

## 4. Conclusions

It was found that the use of bone conduction hearing implants may cause a statistically significant increase in the level of localized SAR values in the head of an implant user—caused by EMF exposure at a frequency of 13.56 MHz emitted from RFID HF readers. The use of such hearing implants may be a contraindication to be near RFID HF readers, especially due to work, while exposed over a long period to an EMF comparable or exceeding exposure limits provided to protect the general public against electromagnetic hazards.

The presented results support the need to assess the EMF hazards to individual implant users exposed to EMF at a level approaching the exposure limits set by international guidelines, with respect to the variability of locations of the head against the EMF source, as well as the type of implant used. Obtained results may be helpful for hearing implant developers and health and safety inspectors.

The mentioned effects of EMF exposure need consideration with respect to the safety of users of hearing implants, also near other sources of high frequency EMF, especially those used in medical centers, for example physiotherapeutic devices, where exposure may locally exceed limits for general public [31].

The possible EMF influence on the electronic structure of the hearing implant also needs to be looked at closely, following the rules of electromagnetic compatibility testing.

## Figures and Tables

**Table 1:** Comparison of the whole head averaged specific energy absorption rate (SAR) (SARwh) under exposure to an EMF at a frequency of 13.56 MHz (emitted by an RFID HF reader) in the head of a hearing implant user and a non-user.

Exposure Scenario (RFID Reader Position)	Dimensions of Numerical Model of Head, Percentile of Polish Male Population	KIwh in User of Implant:	
		Bonebridge	BAHA
a (vertical, plane at ear)	5	**1.10**	1.04
	50	1.01	1.02
	95	0.99	1.01
b (vertical, plane at face)	5	1.00	1.04
	50	1.01	1.01
	95	1.01	0.99
c (horizontal, at ear)	5	1.09	1.06
	50	1.08	1.02
	95	1.08	1.05
d (horizontal, at face)	5	1.02	1.02
	50	1.05	1.02
	95	1.06	1.00
e (vertical, edge at ear)	5	**1.10**	1.10
	50	1.06	1.07
	95	1.00	1.07
f (vertical, edge at face)	5	1.09	**1.11**
	50	1.03	1.01
	95	1.08	0.99
g (vertical, edge at implant)	5	1.02	0.99
	50	1.00	1.04
	95	0.99	1.03

KIwh = SARwh(impl)/SARwh(nu); impl—hearing implant user; nu—non-user; max value underlined.

**Table 2:** Comparison of localized SAR averaged over any 10 g of tissues in the head (SARlh) under exposure to an EMF at a frequency of 13.56 MHz (emitted by an RFID HF reader) in the head of a hearing implant user and a non-user.

Exposure Scenario (RFID Reader Position)	Dimensions of Numerical Model of Head, Percentile of Polish Male Population	KIlh in User of Implant:	
		Bonebridge	BAHA
a (vertical, plane at ear)	5	1.36	1.90
	50	1.21	1.82
	95	1.18	1.61
b (vertical, plane at face)	5	1.00	1.35
	50	1.05	1.33
	95	1.04	1.23
c (horizontal, at ear)	5	1.34	1.75
	50	1.36	1.86
	95	1.40	1.98
d (horizontal, at face)	5	1.22	1.80
	50	1.20	1.74
	95	1.18	1.65
e (vertical, edge at ear)	5	1.49	1.47
	50	1.32	1.43
	95	1.21	1.32
f (vertical, edge at face)	5	1.47	**2.07**
	50	1.36	1.90
	95	1.32	1.81
g (vertical, edge at implant)	5	**1.60**	1.72
	50	1.54	1.67
	95	1.44	1.56

KIlh = SARlh(implant)/SARlh(nu); impl—hearing implant user; nu—non-user; max value underlined.
